# Inpatient rehabilitation therapy in stroke patients with reperfusion therapy: a national prospective registry study

**DOI:** 10.1186/s12883-023-03144-3

**Published:** 2023-04-05

**Authors:** Shengde Li, Yixiu Lu, Shiyuan Fang, Longde Wang, Bin Peng

**Affiliations:** 1 Department of Neurology, State Key Laboratory of Complex Severe and Rare Diseases, Peking Union Medical College Hospital, Peking Union Medical College and Chinese Academy of Medical Sciences, Shuaifuyuan1, Dong Cheng District, Beijing, 100730 China; 2 The General Office of Stroke Prevention Project Committee, National Health Commission of the People’s Republic of China, No. 118, Guang’anmen Inner Street, Beijing, 100053 China

**Keywords:** Stroke rehabilitation, Thrombolysis, Endovascular therapy, Real world, Bigdata

## Abstract

**Background:**

Little is known about the rate of real-world inpatient rehabilitation therapy (IRT) after stroke. We aimed to determine the rate of inpatient rehabilitation therapy and its associated factors in patients who undergo reperfusion therapy in China.

**Methods:**

This national prospective registry study included hospitalized ischemic stroke patients aged 14–99 years with reperfusion therapy between January 1, 2019, and June 30, 2020, collecting hospital-level and patient-level demographic and clinical data. IRT included acupuncture or massage, physical therapy, occupational therapy, speech therapy, and others. The primary outcome was the rate of patients receiving IRT.

**Results:**

We included 209,189 eligible patients from 2191 hospitals. The median age was 66 years, and 64.2% were men. Four in five patients received only thrombolysis, and the rest 19.2% underwent endovascular therapy. The overall rate of IRT was 58.2% (95% CI, 58.0–58.5%). Differences in demographic and clinical variables existed between patients with and without IRT. The rates of acupuncture or massage, physical therapy, occupational therapy, speech therapy, and other rehabilitation interventions were 38.0%, 28.8%, 11.8%, 14.4%, and 22.9%, respectively. The rates of single and multimodal interventions were 28.3% and 30.0%, respectively. A lower likelihood of receiving IRT was associated with being 14–50 or 76–99 years old, female, from Northeast China, from Class-C hospitals, receiving only thrombolysis, having severe stroke or severe deterioration, a short length of stay, Covid-19 pandemic and having intracranial or gastrointestinal hemorrhage.

**Conclusion:**

Among our patient population, the IRT rate was low with limited use of physical therapy, multimodal interventions, and rehabilitation centers and varied by demographic and clinical features. The implementation of IRT remains a challenge for stroke care, warranting urgent and effective national programs to enhance post-stroke rehabilitation and the adherence to guidelines.

**Supplementary Information:**

The online version contains supplementary material available at 10.1186/s12883-023-03144-3.

## Introduction

Stroke is the leading cause of death and disability-adjusted life-years in China with a rapidly increasing incidence[1, 2]. Among 2 million new cases annually, ischemic stroke constitutes 69.6%, with one-third of affected individuals being disabled or dead upon follow-up [2, 3]. To improve the neurological outcome of ischemic stroke, a series of interventions have been recommended by international and Chinese guidelines [2]. Stroke rehabilitation is increasingly regarded as an essential part of stroke care, as it reduces the stroke survivors’ struggles with daily tasks [4, 5]. Early rehabilitation is strongly recommended, and rehabilitation care provided in inpatient rehabilitation facilities is preferred to that provided in skilled nursing facilities or nursing homes for improving functional outcome [4, 6, 7]. Among hospitalized patients, early rehabilitation is performed in the form of inpatient rehabilitation therapy (IRT). Moreover, an increasing number of studies have shown substantial advances in rehabilitation strategies [5, 8]. Based on current samples and retrospective analysis, the overall rates of stroke rehabilitation ranged from 11.5 to 53.0% in China and from 37 to 61% in western countries [9–12]. However, on a large scale, details regarding stroke survivors receiving rehabilitation care and factors associated with such care remain unknown in China.

To improve the national quality of stroke care, the China Stroke Prevention Project Committee (CSPPC Stroke Program) has launched a series of stroke programs with evidence-based support, where the process and quality of stroke care are interactively monitored [10, 13]. A set of best practice strategies and auditing criteria was introduced to hospitals to minimize variation and to standardize care [13].

Reperfusion therapy with additional rehabilitation may be an optimal strategy for acute ischemic stroke (AIS) [7]. However, post-stroke rehabilitation faces tremendous challenges in China, including unavailable insurance, insufficient number of well-trained therapists, poor system for long-term rehabilitation, and underuse of early rehabilitation [14, 15]. And the baseline data were missing, thus, the strategy for optimizing stroke care remained undetermined.

Based on national prospective registry data of the CSPPC Stroke Program, we performed the present study (CSPPC-R) to determine the IRT rate and its associated factors among hospitalized AIS patients with reperfusion therapy.

## Methods

### Data source, Study Design, and Study Population

Issued in 2016 by CSPPC, the Stroke Center Work Plan aims to improve outcomes with evidence-based stroke care and provides high-quality patient-level data of stroke in a real-world setting covering 31 provinces in mainland China, which has been published in detail [10, 13]. All data were collected by trained hospital personnel and monitored by each stroke center, provincial project offices, and the national project committee in real time. The quantity and quality of the reports were verified monthly. Hospitals that failed to pass three consecutive audits were disqualified from the reporting system. The diagnosis of AIS was confirmed by the International Classification of Diseases, Ninth Revision, Clinical Modification, and only patients with intravenous thrombolysis or Endovascular therapy (EVT) were registered in the BOSC (Bigdata Observatory Platform for Stroke of China), according to the study design.

**Fig. 1:**
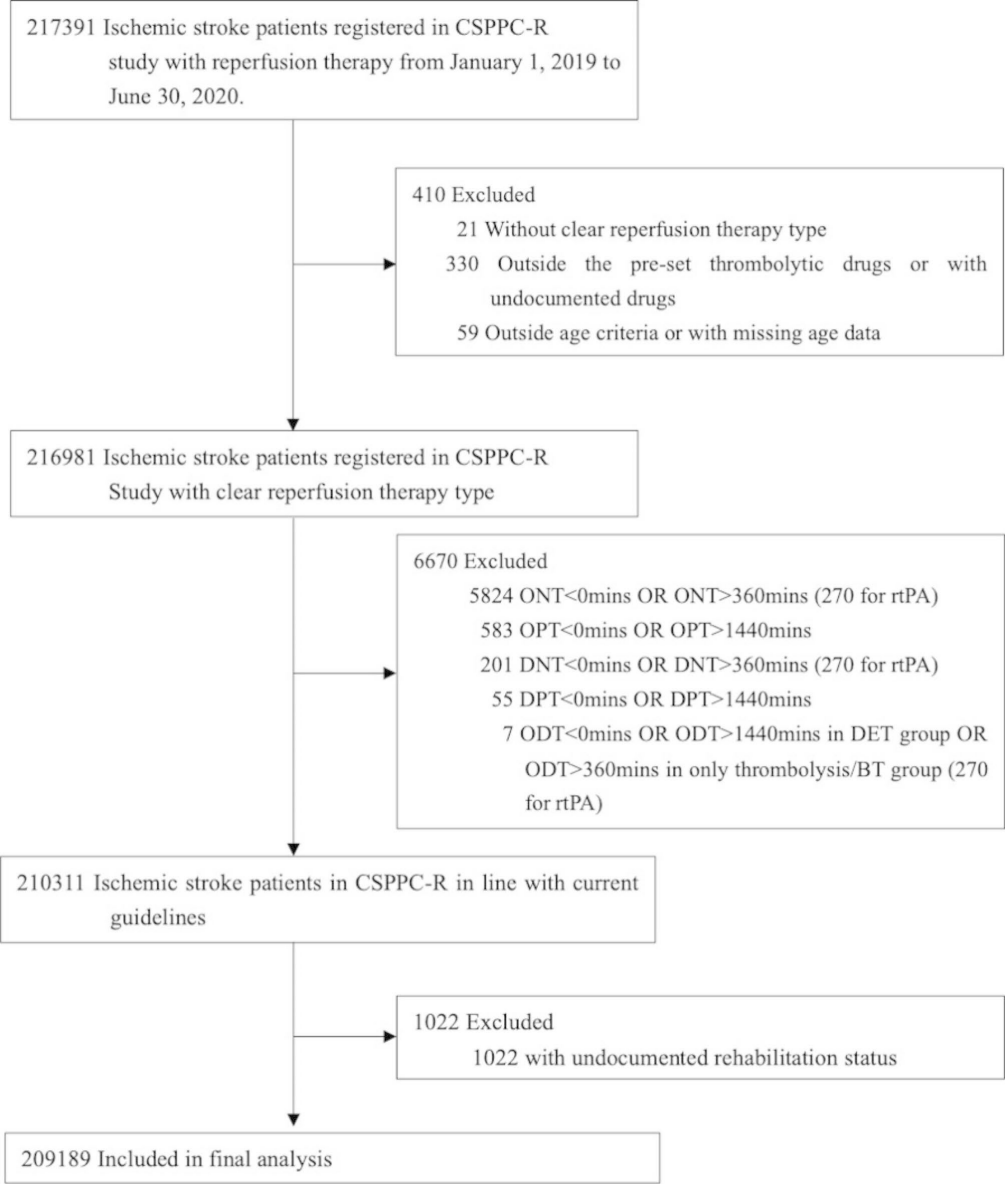
Flow of ischemic patients with reperfusion therapy in CSPPC-R study Abbreviations: BT, bridging thrombolysis; CSPPC: the China Stroke Prevention Project Committee; DET: direct endovascular therapy; DNT: door-to-needle time; DPT: door-to-puncture time; ODT: onset-to-door time; ONT: onset-to-needle time; OPT: onset-to-puncture time; rtPA: recombinant tissue plasminogen activator;

The present CSPPC-R study, with all data derived from the Stroke Center Work Plan (Figure S1 in the Supplement), was approved by the Ethics Committee of Peking Union Medical College Hospital, with a waiver of informed consent (no.: S-K988). The CSPPC-R study inclusion criteria required patients to (1) have been diagnosed with AIS; (2) be aged between 14 and 99 years; (3) have a stroke onset time (if unavailable, the time of initiating reperfusion, the time of hospital arrival or admission were used as alternatives) from January 1, 2019, to June 30, 2020; (4) have received intravenous thrombolysis or EVT; and (5) have received the following thrombolytic drugs: recombinant tissue plasminogen activator (r-tPA), urokinase, anistreplase, reteplase, tenecteplase, or recombinant human pro-urokinase (for those with intravenous thrombolysis). Patients were excluded if they had (1) an unknown reperfusion therapy type; (2) reported time measures against Chinese guidelines, e.g., the onset-to-door time (ONT) > 270 mins for those receiving r-tPA (Fig. 1) [16]; and (3) no documented rehabilitation data.

## Variables

In brief, the reported content of the CSPPC-R study included personal-level and hospital-level demographic and clinical characteristics, details were published elsewhere [10, 13]. The definitions of length of stay (LOS), △NIHSS24h, subgroups of NIHSS score at 24 h, the type of reperfusion therapy, reperfusion time, hospital levels, date of Covid19 pandemic and intracranial hemorrhage were shown in Table S1. △NIHSS24h was calculated by the formula: △NIHSS24h = initial NIHSS score–NIHSS score at 24 h after reperfusion procedure. Then, △NIHSS24h was divided as 4 subgroups: (1) Severe deterioration: △NIHSS24h ≤ -9; (2) Mild deterioration: -8 ≤ △NIHSS24h ≤-1; (3) Stable status, △NIHSS24h = 0; (4) Improvement: △NIHSS24h ≥ 1. Mainly based on the quality of medical care and management, all hospitals in China were graded by the National Health Commission with standard criteria. In our study, hospitals were divided into three levels (details in the Table S1): (1) High-level: class-A hospital; (2) Middle-level: class-B hospital; (3) Low-level: class-C hospital.

## Outcomes

The IRT rate was the primary outcome. IRT includes the following five interventions: (1) traditional rehabilitation (TR): acupuncture or massage [4, 15, 17]; (2) physical therapy (PT); (3) occupational therapy (OT); (4) speech therapy (ST); and (5) other interventions of rehabilitation (OIR): cognitive training, swallowing therapy, psychotherapy, or physiotherapy based on traditional Chinese medicine (TCM). Acupuncture was also recommended by Chinese and international guidelines [4, 15], and many studies showed the efficacy of acupuncture on improving functional outcome after stroke [17]. To better reflect the real-word IRT in China, we included acupuncture/massage as a part of it. In addition, the separate rates of acupuncture/massage, PT, OT, ST, and OIR were showed in our study. The number of rehabilitation interventions was also calculated (range: 0–5) and were divided as single intervention (n = 1) or multimodal interventions (n ≥ 2) based on which the models of IRT was used. The locations for IRT were classified as (1) only bedside, (2) inpatient rehabilitation center (IRC) plus bedside, or (3) only IRC.

## Statistical analyses

Continuous variables with non-normal distributions are presented as median and interquartile ranges and were compared using the Wilcoxon rank-sum test. The Pearson χ^2^ test was used to analyze independent categorical variables, which are presented as frequency and percentage. The overall in-hospital rehabilitation rate is presented as a rate with a corresponding 95% confidence interval (CI).

Continuous variables, including the NIHSS score at 24 h, △NIHSS24h, and LOS, were divided into categorical variables according to guidelines and previous reports [4, 9]. Missing data were not included in the logistic regression. Confounders in logistic model were determined according to the following steps. First, we used univariate logistic regression analysis to investigate the association between IRT and the covariates, including age, sex, nationality, hospital level, BMI, NIHSS score, mRS, LOS and etc. The covariate with P ≥ 0.10 was excluded. Second, the multicollinearity test was conducted by assessing the variance inflation factor and using Pearson correlation coefficient statistic. Stepwise logistic regression analysis was also performed to exclude the covariates with collinearity. Finally, we determined the confounders in the logistic model based on: (1) the screening results of the above-mentioned two steps, and (2) previous reports and clinical practice [9]. Then, we used binary logistic regression models to analyze the factors associated with IRT.

All statistical analyses were two-sided, with a significance level of p < 0.05. The analyses were performed using SAS version 9.3 (SAS Institute Inc.).

## Results

Of 217,391 patients registered in the CSPPC-R study on the BOSC, 3.7% (8102) were excluded according to the enrollment criteria (Fig. 1), and 96.3% (209,189) were included in the final analysis covering 31 provinces and 2191 hospitals in mainland China with relatively limited missing exposure variables (Table S2 in the Supplement). Hospital-level characteristics in different regions are shown in Table S3 and Table S4 in the Supplement. At least half of the patients were enrolled in a class A hospital in every region. The overall ratio of patients receiving EVT to only thrombolysis was 1:4, while ratios were 1:2.5 in Class-A hospitals, 1:3.3 in patients with large artery atherosclerosis stroke, and 1: 1.5 in those with cardioembolic stroke (Table S5 in the Supplement). The median age was 66 years, with 64.2% male, 2.9% minorities, and 51.5% with large artery atherosclerosis stroke. The initial median NIHSS score was 7, and the median 24-hour NIHSS score decreased to 4. The proportion of patients with an initial mRS score ≥ 3 was 38.9%. The median LOS was 9 days. The rates of intracranial and gastrointestinal hemorrhages were 4.3% and 0.6%, respectively.

The overall rate of IRT was 58.2% (95% CI, 58.0–58.5), which varied across regions and patient subgroups (Table 1). Patients received IRT were older and had a higher initial NIHSS score (8 vs. 6), a higher mRS score (2 vs. 1), a higher NIHSS score at 24 h (5 vs. 2), and longer LOS (10 vs. 7 days) than those without IRT.

**Table 1:** Inpatient rehabilitation therapy rates by demographic and clinical variables

	Overall^a^	Without IRT^a^	With IRT^a^	P value ^c^
Total, n (%)	209 189 (100)	87,351 (41.8)	121,838 (58.2)	NA
Age, years ^b^	66 (57–74)	66 (56–74)	66 (57–74)	< 0.0001
Age group, years				< 0.0001
14–50	23,635 (11.3)	10,356 (43.8)	13,279 (56.2)	
51–75	141,877 (67.8)	59,591 (42.0)	82,286 (58.0)	
76–99	43,677 (20.9)	17,404 (39.8)	26,273 (60.2)	
Sex				0.1221
Male	134,396 (64.2)	56,286 (41.9)	78,110 (58.1)	
Female	74,789 (35.8)	31,062 (41.5)	43,727 (58.5)	
Nationality				0.8241
Han	203,038 (97.1)	84,791 (41.8)	118,247 (58.2)	
Minorities	6151 (2.9)	2560 (41.6)	3591 (58.4)	
Region				< 0.0001
Northeast	21,289 (10.2)	11,720 (55.1)	9569 (44.9)	
North	38,551 (18.4)	19,824 (51.4)	18,727 (48.6)	
East	61,773 (29.5)	24,351 (39.4)	37,422 (60.6)	
Central	35,112 (16.8)	13,883 (39.5)	21,229 (60.5)	
South	18,615 (8.9)	5392 (29.0)	13,223 (71.0)	
Southwest	23,698 (11.3)	8283 (35.0)	15,415 (65.0)	
Northwest	10,151 (4.9)	3898 (38.4)	6253 (61.6)	
Hospital level				< 0.0001
Class A	121,492 (58.1)	46,146 (38.0)	75,346 (62.0)	
Class B	27,284 (13.0)	11,865 (43.5)	15,419 (56.5)	
Class C	60,385 (28.9)	29,317 (48.6)	31,068 (51.4)	
BMI				< 0.0001
< 24	104,459 (58.6)	42,274 (40.5)	62,185 (59.5)	
24-	70,558 (39.6)	29,819 (42.3)	40,739 (57.7)	
32-	3291 (1.9)	1531 (46.5)	1760 (53.5)	
TOAST				< 0.0001
LAA	107,722 (51.5)	42,323 (39.3)	65,399 (60.7)	
CE	30,060 (14.4)	11,444 (38.1)	18,616 (61.9)	
SAO	60,755 (29.1)	28,076 (46.2)	32,679 (53.8)	
SOC	1930 (0.9)	801 (41.5)	1129 (58.5)	
SUC	8663 (4.1)	4686 (54.1)	3977 (45.9)	
Reperfusion Therapy				< 0.0001
Only thrombolysis	168,977 (80.8)	74,955 (44.4)	94,022 (55.6)	
BT	11,766 (5.6)	3570 (30.3)	8196 (69.7)	
DET	28,446 (13.6)	8826 (31.0)	19,620 (69.0)	
Reperfusion time				< 0.0001
Early	115,779 (56.5)	49,328 (42.6)	66,451 (57.4)	
Late	89,180 (43.5)	36,104 (40.5)	53,076 (59.5)	
Initial NIHSS score^b^	7 (4–13)	6 (3–12)	8 (4–14)	< 0.0001
Initial mRS^b^	2 (0–4)	1 (0–4)	2 (0–4)	< 0.0001
Initial mRS				< 0.0001
0	59,708 (34.1)	23,880 (40.0)	35,828 (60.0)	
1	26,814 (15.3)	12,711 (47.4)	14,103 (52.6)	
2	20,436 (11.7)	8494 (41.6)	11,942 (58.4)	
3	18,893 (10.8)	7018 (37.1)	11,875 (62.9)	
4	32,477 (18.5)	11,237 (34.6)	21,240 (65.4)	
5	16,902 (9.6)	6724 (39.8)	10,178 (60.2)	
NIHSS score at 24h^b^	4 (1–9)	2 (0–6)	5 (2–10)	< 0.0001
△NIHSS24h^b^	2 (0–4)	2 (0–4)	2 (0–4)	< 0.0001
LOS, days^b^	9 (5–13)	7 (4–11)	10 (7–14)	< 0.0001
ICH				< 0.0001
No	200,123 (95.7)	83,200(41.6)	116,923 (58.4)	
Yes	9028(4.3)	4139(45.9)	4889 (54.1)	
GIH				< 0.0001
No	207,860 (99.4)	86,709 (41.7)	121,151(58.3)	
Yes	1291 (0.6)	630 (48.8)	661 (51.2)	
Covid19 pandemic				0.6708
Before	145,394 (69.5)	60,668 (41.7)	84,726 (58.3)	
During	63,795 (30.5)	26,683 (41.8)	37,112 (58.2)	

Abbreviations: BMI, body mass index; BT, bridging thrombolysis; CE, cardioembolism; DET, direct endovascular therapy; GIH, gastrointestinal hemorrhage; ICH, intracranial hemorrhage; IRT, inpatient rehabilitation therapy; LAA, large artery atherosclerosis; LOS, length of stay; mRS, modified ranking score; NIHSS, National Institutes of Health Stroke Scale; SAO, small artery occlusion; SOC, stroke of other determined cause; SUC, stroke of undetermined cause

^a^ Data are expressed as No. (%) unless otherwise indicated

^b^ Data are presented as median (interquartile range)

^c^ For different subgroups and the use of IRT.

**Fig. 2:**
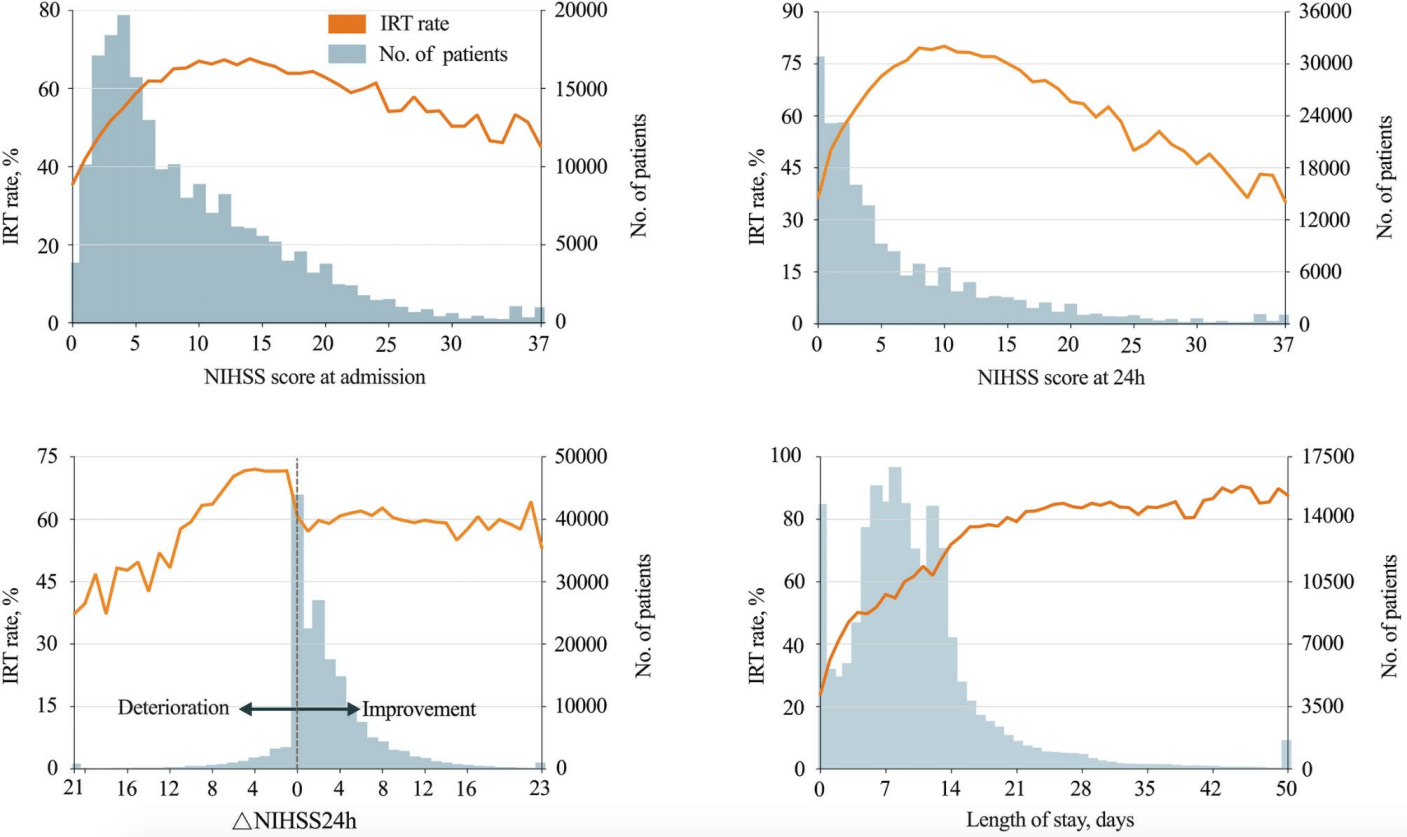
The association between IRT rates and NIHSS score, △NIHSS24h, and length of stay Abbreviations: IRT, inpatient rehabilitation therapy; NIHSS, National Institutes of Health Stroke Scale NIHSS score at admission: 37 for NIHSS score ≥ 37 NIHSS score at 24 h: 37 for NIHSS score ≥ 37 △NIHSS24h: 23 for NIHSS score ≥ 23;21 for NIHSS score ≥ 21 Length of stay: 50 for ≥ 50 days

Figure 2 shows the association between the IRT rate and initial NIHSS score, NIHSS score at 24 h, △NIHSS24h, and LOS. The IRT rate showed an inverted U-shaped relationship with initial NIHSS score, which peaked at 67.7% with a NIHSS score of 14. The inverted U-shaped curve was also detected between IRT rate and 24-hour NIHSS score. The NIHSS scores of 80% of patients were ≤ 15 at baseline and ≤ 11 at 24 h, respectively. According to △NIHSS24h, the IRT rate seemed stable for those with neurological improvement, but it increased with mild neurological deterioration and decreased with severe neurological deterioration. The IRT rate showed a positive correlation with LOS, and most patients (80%) stayed in the hospital for ≤ 13 days (Table S6 in the Supplement).

The rates of TR, PT, OT, ST, and OIR were 38.0% (range: 24.7–65.4%), 28.8% (14.7–52.5%), 11.8% (5.1–17.9%), 14.4% (7.0–26.8%), and 22.9% (11.1–38.2%), respectively and varied in subgroups (Table 2). The overall rate of multimodal interventions was slightly higher than those of the single intervention rate (30.0% vs. 28.3%). In most subgroups, multimodal interventions were used more often than single intervention. Compared with single intervention, the proportion of PT increased markedly among those with multimodal interventions (Figure S2 in the Supplement). Besides, among patients receiving IRT, the overall proportions of rehabilitation locations were 80.0% for only bedside, 12.5% for IRC plus bedside, and 7.5% for only IRC (Table S7 in the Supplement).

**Table 2:** Rates of different rehabilitation interventions and IRT model by demographic and clinical variables

	Rehabilitation intervention ^a^						Model of IRT ^a^	
	TR	PT	OT	ST	OIR		Singe intervention	Multimodal intervention
Overall	79,530 (38.0)	60,228 (28.8)	24,609 (11.8)	30,021 (14.4)	47,906 (22.9)		59,178 (28.3)	62,660 (30.0)
Age group, years								
14–50	8678 (36.7)	6535 (27.7)	2743 (11.6) ^b^	3187 (13.5)	5166 (21.9)		6452 (27.3)	6827 (28.9)
51–75	54,026 (38.1)	40,395 (28.5)	16,584 (11.7)	19,988 (14.1)	31,908 (22.5)		40,343 (28.4)	41,943 (29.6)
76–99	16,826 (38.5)	13,298 (30.5)	5282 (12.1)	6846 (15.7)	10,832 (24.8)		12,383 (28.4)	13,890 (31.8)
Sex								
Male	51,124 (38.0)^b^	38,526 (28.7)^b^	15,739 (11.7)^b^	19,250 (14.3)^b^	30,567 (22.7)		38,037 (28.3)	40,073 (29.8)^b^
Female	28,405 (38.0)	21,701 (29.0)	8869 (11.9)	10,771 (14.4)	17,339 (23.2)		21,141 (28.3)	22,586 (30.2)
Nationality								
Han	77,031 (37.9)	58,419 (28.8)^b^	23,759 (11.7)	28,985 (14.3)	46,371 (22.8)		57,562 (28.4)	60,685 (30.0)
Minority	2499 (40.6)	1809 (29.4)	850 (13.8)	1036 (16.8)	1535 (25.0)		1616 (26.3)	1975 (32.1)
Region								
Northeast	7231 (34.0)	3128 (14.7)	1081 (5.1)	1499 (7.0)	2364 (11.1)		6219 (29.2)	3350 (15.7)
North	11,793 (30.6)	8761 (22.7)	3267 (8.5)	3786 (9.8)	5312 (13.8)		10,412 (27.0)	8315 (21.6)
East	22,563 (36.5)	20,060 (32.5)	8014 (13.0)	9842 (15.9)	13,359 (21.6)		18,468 (29.9)	18,954 (30.7)
Central	13,475 (38.4)	11,010 (31.4)	5325 (15.2)	5544 (15.8)	10,264 (29.2)		9120 (26.0)	12,109 (34.5)
South	9550 (51.3)	6833 (36.7)	2126 (11.4)	3162 (17.0)	6129 (32.9)		5718 (30.7)	7505 (40.3)
Southwest	10,740 (45.3)	7569 (31.9)	3567 (15.1)	4668 (19.7)	7686 (32.4)		6287 (26.5)	9128 (38.5)
Northwest	4178 (41.2)	2867 (28.2)	1229 (12.1)	1520 (15.0)	2792 (27.5)		2954 (29.1)	3299 (32.5)
Hospital type								
Western medicine	73,582 (37.0)	57,428 (28.9)	23,034 (11.6)	28,354 (14.3)	45,377 (22.8)		55,774 (28.0)	59,069 (29.7)
TCM	5774 (57.9)	2712 (27.2)	1551 (15.6)	1629 (16.3)	2468 (24.8)		3321 (33.3)	3487 (35.0)
Hospital level								
Class A	50,229 (41.3)	37,002 (30.5)	14,579 (12.0)	19,042 (15.7)	28,559 (23.5)		36,147 (29.8)	39,199 (32.3)
Class B	10,100 (37.0)	7785 (28.5)	3540 (13.0)	3749 (13.7)	6279 (23.0)		7376 (27.0)	8043 (29.5)
Class C	19,196 (31.8)	15,473 (25.6)	6487 (10.7)	7230 (12.0)	13,067 (21.6)		15,654 (25.9)	15,414 (25.5)
BMI								
< 24	40,272 (38.6)	30,707 (29.4)	12,505 (12.0)	15,864 (15.2)	25,043 (24.0)		30,103 (28.8)	32,082 (30.7)
24-	26,571 (37.7)	19,929 (28.2)	8092 (11.5)	9699 (13.8)	15,631 (22.2)		20,043 (28.4)	20,696 (29.3)
32-	1178 (35.8)	878 (26.7)	369 (11.2)	374 (11.4)	671 (20.4)		840 (25.5)	920 (28.0)
TOAST								
LAA	43,282 (40.2)	33,274 (30.9)	13,893 (12.9)	16,786 (15.6)	26,112 (24.2)		30,837 (28.6)	34,562 (32.1)
CE	12,156 (40.4)	10,058 (33.5)	4007 (13.3)	5294 (17.6)	7769 (25.8)		8178 (27.2)	10,438 (34.7)
SAO	21,055 (34.7)	14,482 (23.8)	5713 (9.4)	6709 (11.0)	11,742 (19.3)		17,537 (28.9)	15,142 (24.9)
SOC	752 (39.0)	536 (27.8)	218 (11.3)	300 (15.5)	481 (24.9)		555 (28.8)	574 (29.7)
SUC	2270 (26.2)	1849 (21.3)	754 (8.7)	924 (10.7)	1793 (20.7)		2065 (23.8)	1912 (22.1)
Reperfusion Therapy								
Only thrombolysis	59,781 (35.4)	45,158 (26.7)	18,384 (10.9)	21,936 (13.0)	36,606 (21.7)		47,507 (28.1)	46,515 (27.5)
BT	5935 (50.4)	4609 (39.2)	2021 (17.2)	2464 (20.9)	3451 (29.3)		3248 (27.6)	4948 (42.1)
DET	13,814 (48.6)	10,461 (36.8)	4204 (14.8)	5621 (19.8)	7849 (27.6)		8423 (29.6)	11,197 (39.4)
Reperfusion time								
Early	42,982 (37.1)	32,747 (28.3)	13,566 (11.7)^b^	16,355 (14.1)	26,155 (22.6)		32,514 (28.1)	33,937 (29.3)
Late	34,968 (39.2)	26,396 (29.6)	10,653 (11.9)	13,071 (14.7)	20,880 (23.4)		25,515 (28.6)	27,561 (30.9)
Initial mRS								
0	22,515 (37.7)	19,343 (32.4)	7413 (12.4)	9091 (15.2)	14,438 (24.2)		16,461 (27.6)	19,367 (32.4)
1	9042 (33.7)	5831 (21.8)	2497 (9.3)	3261 (12.2)	5190 (19.4)		7854 (29.3)	6249 (23.3)
2	7858 (38.5)	5230 (25.6)	2272 (11.1)	2764 (13.5)	4622 (22.6)		6227 (30.5)	5715 (28.0)
3	7999 (42.3)	5723 (30.3)	2531 (13.4)	3132 (16.6)	4725 (25.0)		5575 (29.5)	6300 (33.4)
4	14,251 (43.9)	11,065 (34.1)	4765 (14.7)	5735 (17.7)	9075 (27.9)		9400 (28.9)	11,840 (36.5)
5	6951 (41.1)	5251 (31.1)	2263 (13.4)	2830 (16.7)	4474 (26.5)		4503 (26.6)	5675 (33.6)
Initial NIHSS score								
0–4	20,535 (29.6)	14,312 (20.6)	5720 (8.3)	6711 (9.7)	12,894 (18.6)		19,581 (28.3)	14,527 (21.0)
5–20	51,358 (42.9)	40,333 (33.7)	16,550 (13.8)	20,340 (17.0)	30,383 (25.4)		34,579 (28.9)	42,128 (35.2)
≥21	6631 (38.6)	5071 (29.5)	2120 (12.4)	2708 (15.8)	4186 (24.4)		4165 (24.3)	5466 (31.8)
NIHSS score at 24 h								
0–4	33,313 (31.1)	24,045 (22.5)	9504 (8.9)	11,866 (11.1)	21,313 (19.9)		30,781 (28.7)	24,579 (22.9)
5–20	37,110 (53.1)	29,547 (42.3)	12,606 (18.0)	15,148 (21.7)	21,149 (30.3)		21,432 (30.7)	31,311 (44.8)
≥21	3826 (34.5)	2869 (25.9)	1141 (10.3)	1363 (12.3)	2518 (22.7)		2651 (23.9)	3032 (27.3)
△NIHSS24h								
Severe deterioration	1259 (31.9)	1014 (25.7)	401 (10.2)	467 (11.8)	852 (21.6)		907 (23.0)	1025 (26.0)
Mild deterioration	7175 (49.7)	5935 (41.1)	2586 (17.9)	2571 (17.8)	4166 (28.9)		4085 (28.3)	6156 (42.7)
Stable	18,021 (41.0)	13,037 (29.7)	5372 (12.2)	6375 (14.5)	10,273 (23.4)		13,065 (29.7)	13,638 (31.0)
Improvement	47,550 (38.0)	36,328 (29.1)	14,818 (11.9)	18,882 (15.1)	29,550 (23.6)		36,582 (29.3)	37,948 (30.4)
LOS, days								
< 7	17,129 (24.7)	13,249 (19.1)	4494 (6.5)	6811 (9.8)	11,709 (16.9)		16,463 (23.8)	13,125 (18.9)
7–20	52,021 (42.1)	38,695 (31.3)	15,826 (12.8)	18,992 (15.4)	30,186 (24.4)		38,330 (31.0)	40,529 (32.8)
≥ 21	9989 (65.4)	8021 (52.5)	4160 (27.2)	4089 (26.8)	5831 (38.2)		4116 (27.0)	8730 (57.1)
ICH								
No	76,203 (38.1)	57,533 (28.8)	23,516 (11.8)^b^	28,705 (14.3)^b^	45,912 (22.9)		57,022 (28.5)	59,901 (29.9)
Yes	3310 (36.7)	2687 (29.8)	1087 (12.0)	1313 (14.5)	1985 (22.0)		2138 (23.7)	2751 (30.5)
GIH								
No	79,106 (38.1)	59,860 (28.8)^b^	24,463 (11.8)^b^	29,856 (14.4)^b^	47,611 (22.9)^b^		58,855 (28.3)	62,296 (30.0)
Yes	407 (31.5)	360 (27.9)	140 (10.8)	162 (12.6)	286 (22.2)		305 (23.6)	356 (27.6)
Covid19 pandemic								
Before	54,649 (37.6)	41,418 (28.5)	16,942 (11.7)	20,370 (14.0)	32,862 (22.6)		41,994 (28.9)	42,732 (29.4)
During	24,881 (39.0)	18,810 (29.5)	7667 (12.0)	9651 (15.1)	15,044 (23.6)		17,184 (26.9)	19,928 (31.2)

Abbreviations: BMI, body mass index; BT, bridging thrombolysis; CE, cardioembolism; DET, direct endovascular therapy; GIH, gastrointestinal hemorrhage; ICH, intracranial hemorrhage; IRT, inpatient rehabilitation therapy; LAA, large artery atherosclerosis; LOS, length of stay; mRS, modified ranking score; NIHSS, National Institutes of Health Stroke Scale; OIR, other interventions of rehabilitation; OT, occupational therapy; PT, physical therapy; SAO, small artery occlusion; SOC, stroke of other determined cause; ST, speech therapy; SUC, stroke of undetermined cause; TCM: traditional Chinese medicine; TR, traditional rehabilitation

^a^ Data are expressed as No. (%)

^b^ P value > 0.05, those without b indicates P value < 0.05. P values are for subgroups and specific intervention, for example, different age groups and the use of TR, different age groups and the use of PT.

**Fig. 3:**
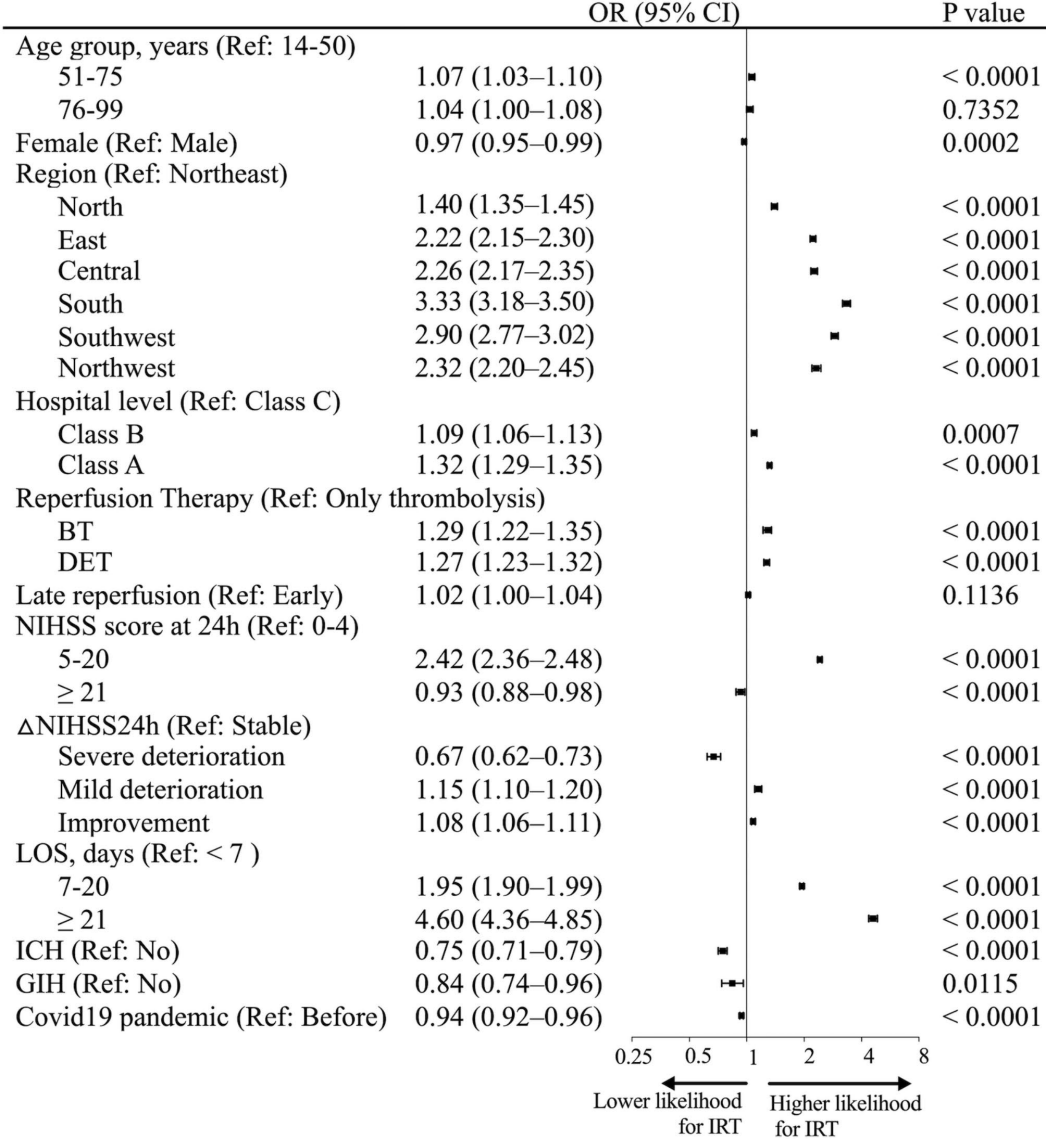
Logistic regression model of variable associated with inpatient rehabilitation therapy Abbreviations: BT, bridging thrombolysis; CI, confidence interval; DET, direct endovascular therapy; GIH, gastrointestinal hemorrhage; ICH, intracranial hemorrhage; LOS, length of stay; NIHSS, National Institutes of Health Stroke Scale; OR, odds ratio

Figure 3 shows the variables independently associated with IRT. Longer LOS and higher-level hospitals were strongly associated with IRT. IRT did not differ between men and women. Those who only received thrombolysis had a significantly lower odds ratio for IRT, but the interval from onset to reperfusion did not affect IRT. In addition, patients with mild or severe stroke or severe deterioration had a decreased odds ratio for IRT, as well as those with intracranial or gastrointestinal hemorrhage. The Covid 19 pandemic reduced the chances of using IRT. The sensitivity analysis showed that 1-day increase in LOS (adjusted OR, 1.069 [95% CI, 1.067–1.071]) and one-grade increase in hospital level (adjusted OR, 1.15 [95% CI, 1.14–1.17]) were significantly associated with IRT (Table S8 in the Supplement). Moreover, sensitivity analysis confirmed the associations between variables and IRT, as shown in Fig. 3.

## Discussion

This nationwide prospective registry study was the first to identify the real-world rate of IRT in patients with ischemic stroke undergoing reperfusion therapy. The rehabilitation evaluation rates were 58.8% in China and 76.1–77.8% in America, which are higher than the IRT rate reported in our study [3, 18]. However, rehabilitation evaluation was not the same as rehabilitation therapy, where a substantial gap existed. Rehabilitation evaluation indicated that AIS patients were assessed by physician, but did not mean that the patients received rehabilitation therapy. A retrospective study in America showed that 61.5% of ischemic stroke patients received PT and OT, which was markedly lower than the rehabilitation evaluation rate [12]. Although rehabilitation has been recommended for stroke recovery and advanced in practice [4, 5], nearly half of hospitalized patients did not receive IRT in our study, with varied rates in the subgroups. For instance, the IRT rate was much lower in those with gastrointestinal hemorrhage (51.2%). Therefore, acute management of ischemic stroke is suboptimal in China. A substantial gap existed between guidelines and clinical practice on the post-stroke rehabilitation in China.

Moreover, the rates of inpatient post-stroke PT in Australia and Norway were 92.9% and 78.6%, respectively, while the rate of PT in our study was 28.8% [11]. The rates of inpatient post-stroke OT were 82.5%, 76.6%, and 11.8% in Australia, Norway and our study, respectively [11]. In the United States, 85.2% of ischemic stroke patients received hospital-based rehabilitation including PT and OT [12]. PT and OT were both underused in China. Even acupuncture/massage was included as a part of IRT, the overall rate of IRT in actual situation was still much lower than that in western countries (58.2% vs. 76.6-85.2%) (5). The differences in definition, content, and amount of PT and OT might exist and generate bias to the real-world rates of IRT. Unfortunately, these data were unavailable in our study nor in others [11, 12].

Although northeast China has the highest incidence and mortality rates of stroke with higher disability-adjusted life-years [1, 19], the IRT rate was the lowest in China. Additionally, approximately three-fourths of patients were treated in class A hospitals in both areas, but the likelihood of IRT in northeast China was less than one-third of that in South China, probably due to its low density of stroke center [2].

Unsurprisingly, the IRT rate increased with LOS in our study [12]. However, we noticed that approximately one-third and four-fifths of patients stayed in the hospital for less than 1 and 2 weeks, respectively, which were shorter than the reported LOS in China (median: 13 days) and longer than that reported in other countries (median: 4 days) [3, 20]. The intervals from stroke onset to IRT varied in clinical trials and practice, and were unclear in the guidelines [4, 15, 21]. Herein, less than half of the patients received IRT within 7 days after admission, and less than one-fifth received PT. The challenge is to increase the rate and quality of IRT in patients with a short LOS. Thus, the implementation of a stroke rehabilitation program is warranted in the first 2 weeks after admission to increase the benefits [22].

Our study confirmed that lower-level hospitals were associated with lower rates of IRT; however, more than two-fifths of patients with ischemic stroke were first-treated in class B or C hospitals [12, 23]. Some lower-level hospitals in China did not have stroke or rehabilitation centers, making a multidisciplinary team for stroke care unavailable [15, 21].

Clinical features were shown to affect IRT. Chinese guidelines recommend starting rehabilitation therapy within 24 h after stroke onset in patients with mild or moderate stroke or with stable neurologic function. Our study found that patients with moderate stroke or mild deterioration had the highest likelihood of IRT, which is different from previous reports [24, 25].This finding suggests that IRT is underused in Chinese patients with minor stroke or improved neurologic function, suggesting further programs to meet the need.

For ischemic stroke patients with reperfusion therapy, IRT is an effective part of the continuum of stroke care for further improved outcomes [6, 7, 22]. In our study, the main intervention of IRT was acupuncture or massage. Rates of PT and OT were low, and rehabilitation centers were not commonly used. Most interventions were only performed at bedside. Thus, the overall contents and structures of IRT in clinical practice were different and weakened according to guidelines and clinical studies [4, 15, 21]. The use of PT and OT in China was substantially lower than that in America [12]. High-quality IRT is another goal of stroke care in China.

Another concern of post-stroke rehabilitation was cost or medical insurance, as rehabilitation was the major contributor to high post-stroke care costs [4, 26]. In China, the mean inpatient cost for ischemic stroke was $2757, and 24.1% of the cost was out-of-pocket spending; the median rehabilitation cost may be no more than tens of dollars [27, 28]. Thus, the cost was probably not the main reason for the lack of IRT, and we postulate high cost-effectiveness for the implementation of IRT in China.

Researchers have noticed the challenge of stroke care during the COVID-19 pandemic, and also suggested early rehabilitation for AIS patients [29]. In China, our study showed that the impact of COVID-19 pandemic on the use of IRT seemed not noteworthy in clinical practice.

Our study presented a substantial gap of stroke rehabilitation between guidelines and real-world, and found possible factors influencing the implement of IRT, which might be ignored by neurologists and policy-makers [14]. Further, this gap might also exist in other countries [11, 12]. How to increase the adherence to stroke rehabilitation guidelines might be another challenge of stroke care. For future studies aiming to promote post-stroke rehabilitation and reduce post-stroke disabilities, our study provides clues and baseline data for study design. Thus, the significance of our study is probably not limited within China [21].

This study has several limitations. First, the CSPPC-R only included hospitalized patients who underwent reperfusion therapy. The data of outpatient or home-based rehabilitation and those without reperfusion therapy or with hemorrhagic stroke remain unknown [30]. Second, the intensity, duration, and time to start IRT were not recorded in our study. Thus, the quality of IRT was unclear and may be heterogeneous, which was another unmet challenge in Chinese stroke care [14, 21]. Third, direct reasons for refusing IRT and patients’ socioeconomic status were not investigated in our study. However, as discussed above, the economic status may not be a barrier to IRT. Fourth, approximately 25,080 (12.0%) patients were excluded from the multivariable analysis, which may have generated selection bias. Besides, unmeasured confounders may have affected the use of IRT, such as socioeconomic status and medical insurance, but were not documented in our study. Finally, our study did not analyze the association between IRT and outcome, as this article is a survey of the actual situation. The results should be interpreted cautiously when applied to other countries.

## Conclusion

Among hospitalized Chinese patients with ischemia undergoing reperfusion therapy, the IRT rate was low with limited use of PT, OT, multimodal interventions, and rehabilitation centers and varied by demographic and clinical features. The implementation of IRT remains a challenge for stroke care, warranting urgent and effective national programs to enhance post-stroke rehabilitation and the adherence to guidelines.

## Electronic supplementary material

Below is the link to the electronic supplementary material.

Supplementary Material 1

## Data Availability

The datasets used and/or analyzed during the current study are available from the corresponding author on reasonable request.

## Abbreviations

AIS: Acute ischemic stroke
BMI: Body mass index
BT: Bridging thrombolysis
CE: Cardioembolism
CSPPC: The China Stroke Prevention Project Committee
CI: Confidence interval
DET: Direct endovascular therapy
DNT: Door-to-needle time
DPT: Door-to-puncture time
EVT: Endovascular therapy
GIH: Gastrointestinal hemorrhage
ICH: Intracranial hemorrhage
IRT: Inpatient rehabilitation therapy
LAA: Large artery atherosclerosis
LOS: Length of stay
mRS: Modified ranking score
NIHSS: National Institutes of Health Stroke Scale
ODT: Onset-to-door time
ONT: Onset-to-needle time
OPT: Onset-to-puncture time
OR: Odds ratio
OT: Occupational therapy
PT: Physical therapy
ST: Speech therapy
TCM: Traditional Chinese medicine
TR: Traditional rehabilitation
rtPA: Recombinant tissue plasminogen activator
SAO: Small artery occlusion
SOC: Stroke of other determined cause
SUC: Stroke of undetermined cause
