# Supplementary material for: Inpatient rehabilitation therapy in stroke patients with reperfusion therapy: a national prospective registry study
Source: BMC Neurol. 2023 Apr 5;23:146. doi: 10.1186/s12883-023-03144-3 (PMC10073784; doi:10.1186/s12883-023-03144-3)
Supplement: Supplementary file 1 — Supplementary Material 1 [file 12883_2023_3144_MOESM1_ESM.docx]

Supplementary Online Content

TableS1. Definitions of key variables in CSPPC-R study.

TableS2. Missing data of exposure variables

TableS3. Number of patients in different hospitals and regions of China

TableS4. Number of hospitals in different regions of China

TableS5. Types of reperfusion therapy varied by hospital levels and ischemic stroke subtype (TOAST)

TableS6. Inpatient rehabilitation therapy rates by NIHSS score, length of stay, hospital type and Systolic pressure at admission

TableS7. Proportions of rehabilitation locations in patients receiving IRT

TableS8. Sensitivity analysis: logistic regression models of variables associated with ITR

FigureS1. Data source of CSPPC-R study

FigureS2. Data of rehabilitation interventions and its numbers

Table S1. Definitions of key variables in CSPPC-R study.

| Variable | Definition | Note |
| --- | --- | --- |
| △NIHSS24h | △NIHSS24h=initial NIHSS score–NIHSS score at 24 hours after reperfusion procedure | NA |
| The NIHSS score at 24 hours: 3 groups | (1) minor stroke, 0–4;  (2) moderate stroke, 5–20;  (3) severe stroke, ≥21. | NA |
| The △NIHSS24h value: 4 subgroups | (1) severe deterioration, △NIHSS24h ≤-9;  (2) mild deterioration, -8≤△NIHSS24h≤-1;  (3) stable status, △NIHSS24h=0;  (4) improvement, △NIHSS24h ≥1. | NA |
| The type of reperfusion therapy: 3 groups | (1) only thrombolysis, patients receiving thrombolysis without EVT;  (2) bridging thrombolysis (BT), patients with thrombolysis followed by EVT;  (3) direct EVT (DET), | NA |
| The reperfusion time: 2 groups | (1) early reperfusion, ONT ≤180 minutes in the only thrombolysis/BT group or OPT ≤270 minutes in the DET group;  (2) late reperfusion, ONT >180 minutes in the only thrombolysis/BT group or OPT >270 minutes in the DET group. | NA |
| Hospital levels: 3 levels | (1) class A: tertiary A hospitals;  (2) class B: tertiary B or tertiary C or undetermined tertiary hospitals;  (3) class C: secondary and primary hospitals. | All hospitals in China were graded by the National Health Commission with standard criteria as primary, secondary, and tertiary hospitals, and A-B-C sub-levels in each group, mainly based on the quality of medical care and management. Tertiary A represents the highest level in this grading system. |
| Covid19 pandemic: 2 periods | (1)“Before Covid19 pandemic” covered date from January 1, 2019 to January 22, 2020;  (2)“During Covid19 pandemic” covered date from January 23, 2020 to June 30, 2020. | NA |
| Length of stay (LOS) | For in-hospital stroke, LOS was calculated as follows: the date of discharge–the date of stroke onset. | NA |
| Intracranial hemorrhage | It was reported after confirmation of computed tomography results and neurological findings, including symptomatic and asymptomatic events. | NA |

TableS2. Missing data of exposure variables

|  | Number | Percentage (%) |
| --- | --- | --- |
| Age | 0 | 0 |
| Sex | 4 | 0 |
| Nationality | 0 | 0 |
| Region | 0 | 0 |
| Hospital level | 28 | 0 |
| Hospital type | 224 | 0.1 |
| Systolic pressure at admission | 53 | 0 |
| BMI | 30881 | 14.8 |
| TOAST | 59 | 0 |
| Reperfusion Therapy | 0 | 0 |
| Reperfusion time | 4230 | 2 |
| Initial NIHSS score | 3116 | 1.5 |
| Initial mRS | 33959 | 16.2 |
| NIHSS score at 24h | 21071 | 10.1 |
| △NIHSS24h | 21820 | 10.4 |
| Length of stay | 876 | 0.4 |
| Intracranial hemorrhage | 38 | 0 |
| Gastrointestinal hemorrhage | 38 | 0 |
| Rehabilitation place | 1604 | 0.8 |
| Covid19 pandemic | 0 | 0 |

Abbreviations: BMI, body mass index; TCM, traditional Chinese medicine.

TableS3. Number of patients in different hospitals and regions of China

|  | Hospital level, No. (%) | | | P value |
| --- | --- | --- | --- | --- |
|  | Class A | Class B | Class C |  |
| Northeast | 15872 (74.6) | 2723 (12.8) | 2694 (12.6) | <0.0001 |
| North | 19530 (50.7) | 3496 (9.1) | 15525 (40.3) |  |
| East | 36207 (58.6) | 11314 (18.3) | 14252 (23.1) |  |
| Central | 17810 (50.8) | 1956 (5.6) | 15318 (43.7) |  |
| South | 14136 (75.9) | 2730 (14.7) | 1749 (9.4) |  |
| Southwest | 11856 (50.0) | 3716 (15.7) | 8126 (34.3) |  |
| Northwest | 6081 (59.9) | 1349 (13.3) | 2721 (26.8) |  |

TableS4. Number of hospitals in different regions of China

|  | Hospital level, No. | | | Total ^a^ |
| --- | --- | --- | --- | --- |
|  | Class A | Class B | Class C |  |
| Northeast | 65 | 25 | 40 | 130 |
| North | 103 | 32 | 240 | 375 |
| East | 207 | 120 | 248 | 575 |
| Central | 102 | 34 | 172 | 308 |
| South | 106 | 36 | 45 | 187 |
| Southwest | 114 | 60 | 231 | 405 |
| Northwest | 74 | 18 | 118 | 210 |

^a^1 hospital with unknow level.

Table S5. Types of reperfusion therapy varied by hospital levels and ischemic stroke subtype (TOAST)

|  | Types of reperfusion therapy, No. (%) | | |
| --- | --- | --- | --- |
|  | Only thrombolysis | BT | DET |
| Hospital level ^a^ |  |  |  |
| Class A | 87245 (71.8) | 8995 (7.4) | 25252 (20.8) |
| Class B | 23590 (86.5) | 1679 (6.1) | 2015 (7.4) |
| Class C | 58115 (96.2) | 1091 (1.8) | 1179 (2.0) |
| TOAST ^b^ |  |  |  |
| LAA | 82968 (77.0) | 7289 (6.8) | 17465 (16.2) |
| CE | 18406 (61.2) | 3529 (11.7) | 8125 (27.0) |
| SAO | 58969 (97.1) | 459 (0.8) | 1327 (2.2) |
| SOC | 1291 (66.9) | 148 (7.7) | 491 (25.4) |
| SUC | 7293 (84.2) | 341 (3.9) | 1029 (11.9) |

Abbreviations: BT, bridging thrombolysis; CE, cardioembolism; DET, direct endovascular therapy; LAA, large artery atherosclerosis; LOS, length of stay; SAO, small artery occlusion; SOC, stroke of other determined cause; SUC, stroke of undetermined cause.

^a^ Missing data was 28;

^b^ Missing data was 59.

TableS6. Inpatient rehabilitation therapy rates by NIHSS score, length of stay, hospital type and Systolic pressure at admission

|  | Overall^a^ | Without IRT^a^ | With IRT^a^ | P value |
| --- | --- | --- | --- | --- |
| Initial NIHSS score |  |  |  | <0.0001 |
| 0-4 | 69312 (33.6) | 35204 (50.8) | 34108 (49.2) |  |
| 5-20 | 119596 (58.0) | 42889 (35.9) | 76707 (64.1) |  |
| ≥21 | 17165 (8.3) | 7534 (43.9) | 9631 (56.1) |  |
| NIHSS score at 24h |  |  |  | <0.0001 |
| 0-4 | 107112 (56.9) | 51572 (48.3) | 55360 (51.7) |  |
| 5-20 | 69915 (37.2) | 17172 (24.6) | 52743 (75.4) |  |
| ≥21 | 11091 (5.9) | 5408 (48.8) | 5683 (51.2) |  |
| △NIHSS24h |  |  |  | <0.0001 |
| Severe deterioration | 3947 (2.1) | 2015 (51.1) | 1932 (48.9) |  |
| Mild deterioration | 14430 (7.7) | 4189 (29.0) | 10241 (71.0) |  |
| Stable | 43956 (23.5) | 17253 (39.3) | 26703 (60.7) |  |
| Improvement | 125036 (66.7) | 50506 (40.4) | 74530 (59.6) |  |
| Length of stay, days |  |  |  | <0.0001 |
| < 7 | 69316 (33.3) | 39728 (57.3) | 29588 (42.7) |  |
| 7-20 | 123714 (59.4) | 44855 (36.3) | 78859 (63.7) |  |
| ≥21 | 15283 (7.3) | 2437 (16.0) | 12846 (84.0) |  |
| Hospital type |  |  |  | <0.0001 |
| Western medicine | 198995 (95.2) | 84152 (42.3) | 114843 (57.7) |  |
| TCM | 9970 (4.8) | 3162 (31.7) | 6808 (68.3) |  |
| Systolic pressure at admission, mmHg |  |  |  | <0.0001 |
| <90 | 333（0.2%） | 175 (52.6) | 158 (47.4) |  |
| 90- | 195636 (93.5) | 81792 (41.8) | 113844 (58.2) |  |
| 190- | 13167 (6.3) | 5366 (40.8) | 7801 (59.2) |  |

Abbreviations: IRT, inpatient rehabilitation therapy; LOS, length of stay; NIHSS, National Institutes of Health Stroke Scale; TCM, traditional Chinese medicine.

^a^ Data are expressed as No. (%)

TableS7. Proportions of rehabilitation locations in patients receiving IRT

|  | Only bedside^a^ | IRC  plus bedside^a^ | Only IRC^a^ | P value |
| --- | --- | --- | --- | --- |
| Overall | 96208 (80.0) | 15073 (12.5) | 8953 (7.5) |  |
| Age group |  |  |  | <0.0001 |
| 14-50 | 10237 (78.2) | 1729 (13.2) | 1134 (8.7) |  |
| 51-75 | 64579 (79.5) | 10355 (12.8) | 6277 (7.7) |  |
| 76-99 | 21392 (82.5) | 2989 (11.5) | 1542 (6.0) |  |
| Sex |  |  |  | <0.0001 |
| Male | 61422 (79.7) | 9736 (12.6) | 5921 (7.7) |  |
| Female | 34785 (80.6) | 5337 (12.4) | 3032 (7.0) |  |
| Nationality |  |  |  |  |
| Han | 93483 (80.1) | 14638 (12.6) | 8554 (7.3) |  |
| Minorities | 2725 (76.6) | 435 (12.2) | 399 (11.2) |  |
| Region |  |  |  | <0.0001 |
| Northeast | 7751 (82.8) | 600 (6.4) | 1014 (10.8) |  |
| North | 14572 (78.9) | 2089 (11.3) | 1800 (9.8) |  |
| East | 30717 (83.4) | 4281 (11.6) | 1854 (5.0) |  |
| Central | 16043 (76.2) | 3658 (17.4) | 1352 (6.4) |  |
| South | 10269 (78.6) | 1664 (12.7) | 1140 (8.7) |  |
| Southwest | 12047 (79.0) | 1951 (12.8) | 1251 (8.2) |  |
| Northwest | 4809 (77.8) | 830 (13.4) | 542 (8.8) |  |
| Hospital level |  |  |  | <0.0001 |
| Class A | 60777 (81.7) | 8973 (12.1) | 4665 (6.3) |  |
| Class B | 11794 (77.5) | 2163 (14.2) | 1256 (8.3) |  |
| Class C | 23635 (77.2) | 3936 (12.9) | 3030 (9.9) |  |
| Hospital type |  |  |  | <0.0001 |
| Western medicine | 91000 (80.2) | 13896 (12.3) | 8504 (7.5) |  |
| TCM | 5068 (76.1) | 1149 (17.3) | 442 (6.6) |  |
| BMI |  |  |  | 0.0128 |
| < 24 | 49621 (80.6) | 7436 (12.1) | 4485 (7.3) |  |
| 24- | 32181 (79.8) | 5075 (12.6) | 3077 (7.6) |  |
| 32- | 1370 (79.0) | 223 (12.9) | 140 (8.1) |  |
| Reperfusion Therapy |  |  |  | <0.0001 |
| Only thrombolysis | 74469 (80.3) | 11129 (12.0) | 7109 (7.7) |  |
| BT | 6320 (77.8) | 1303 (16.0) | 500 (6.2) |  |
| DET | 15419 (79.5) | 2641 (13.6) | 1344 (6.9) |  |
| Reperfusion time |  |  |  | 0.0468 |
| Early | 52461 (80.0) | 8344 (12.7) | 4748 (7.2) |  |
| Late | 42015 (80.2) | 6464 (12.3) | 3926 (7.5) |  |
| Initial mRS |  |  |  | <0.0001 |
| 0 | 28621 (81.3) | 4599 (13.1) | 1983 (5.6) |  |
| 1 | 11327 (81.2) | 1550 (11.1) | 1066 (7.7) |  |
| 2 | 9418 (79.7) | 1382 (11.7) | 1016 (8.6) |  |
| 3 | 9209 (78.3) | 1547 (13.2) | 1002 (8.5) |  |
| 4 | 16470 (78.1) | 2970 (14.1) | 1635 (7.8) |  |
| 5 | 8125 (80.4) | 1398 (13.8) | 580 (5.7) |  |
| Initial NIHSS score |  |  |  | <0.0001 |
| 0-4 | 27475 (81.7) | 3600 (10.7) | 2537 (7.6) |  |
| 5-20 | 59867 (79.0) | 10159 (13.4) | 5729 (7.6) |  |
| ≥21 | 7830 (82.1) | 1192 (12.5) | 520 (5.4) |  |
| NIHSS score at 24h |  |  |  | <0.0001 |
| 0-4 | 44924 (82.2) | 5828 (10.7) | 3883 (7.1) |  |
| 5-20 | 40411 (77.5) | 7747 (14.9) | 3991 (7.6) |  |
| ≥21 | 4795 (84.9) | 624 (11.0) | 230 (4.1) |  |
| △NIHSS24h |  |  |  | <0.0001 |
| Severe deterioration | 1592 (83.1) | 221 (11.5) | 103 (5.4) |  |
| Mild deterioration | 7763 (76.6) | 1612 (15.9) | 765 (7.5) |  |
| Stable | 20990 (79.6) | 3389 (12.8) | 2003 (7.6) |  |
| Improvement | 59473 (80.8) | 8943 (12.1) | 5207 (7.1) |  |
| LOS, days |  |  |  | <0.0001 |
| < 7 | 25300 (86.6) | 2376 (8.1) | 1535 (5.3) |  |
| 7-20 | 62523 (80.4) | 9512 (12.2) | 5729 (7.4) |  |
| ≥21 | 7981 (62.7) | 3110 (24.4) | 1640 (12.9) |  |
| ICH |  |  |  | <0.0001 |
| No | 92180 (79.9) | 14500 (12.6) | 8695 (7.5) |  |
| Yes | 4009 (82.9) | 570 (11.8) | 255 (5.3) |  |
| GIH |  |  |  | 0.0414 |
| No | 95647 (80.0) | 14995 (12.5) | 8917 (7.5) |  |
| Yes | 542 (83.4) | 75 (11.5) | 33 (5.1) |  |
| Covid19 pandemic |  |  |  | <0.0001 |
| Before | 67209 (80.1) | 10208 (12.2) | 6515 (7.8) |  |
| During | 28999 (79.9) | 4865 (13.4) | 2438 (6.7) |  |

Abbreviations: BMI, body mass index; BT, bridging thrombolysis; DET, direct endovascular therapy; GIH, gastrointestinal hemorrhage; ICH, intracranial hemorrhage; IRT, inpatient rehabilitation therapy; IRC: inpatient rehabilitation center; LOS, length of stay; mRS, modified ranking score; NIHSS, National Institutes of Health Stroke Scale; TCM, traditional Chinese medicine.

TableS8. Sensitivity analysis: logistic regression models of variables associated with ITR

|  | OR (95% CI) | P value |
| --- | --- | --- |
| Age group |  |  |
| 14-50 | 1 [Reference] |  |
| 51-75 | 1.07 (1.04–1.11) | <0.0001 |
| 76-99 | 1.04 (1.00–1.08) | 0.8125 |
| Sex |  |  |
| Male | 1 [Reference] |  |
| Female | 0.97 (0.95–0.99) | 0.0033 |
| Region |  |  |
| Northeast | 1 [Reference] |  |
| North | 1.35 (1.30–1.40) | <0.0001 |
| East | 2.15 (2.08–2.23) | <0.0001 |
| Central | 2.15 (2.07–2.24) | <0.0001 |
| South | 3.14 (3.00–3.30) | <0.0001 |
| Southwest | 2.81 (2.69–2.94) | <0.0001 |
| Northwest | 2.28 (2.16–2.41) | <0.0001 |
| Hospital level, per grade increment | 1.15 (1.14–1.17) | <0.0001 |
| Reperfusion Therapy |  |  |
| Only thrombolysis | 1 [Reference] |  |
| BT | 1.25 (1.18–1.31) | <0.0001 |
| DET | 1.24 (1.20–1.29) | <0.0001 |
| Reperfusion time |  |  |
| Early | 1 [Reference] |  |
| Late | 1.02 (1.00–1.04) | 0.0458 |
| NIHSS score at 24h |  |  |
| 0-4 | 1 [Reference] |  |
| 5-20 | 2.35 (2.29–2.40) | <0.0001 |
| ≥21 | 0.89 (0.84–0.93) | <0.0001 |
| △NIHSS24h |  |  |
| Severe deterioration | 0.68 (0.63–0.74) | <0.0001 |
| Mild deterioration | 1.15 (1.10–1.20) | <0.0001 |
| Stable | 1 [Reference] |  |
| Improvement | 1.09 (1.06–1.12) | <0.0001 |
| Length of stay, per day increment | 1.069 (1.067–1.071 ) | <0.0001 |
| Intracranial hemorrhage |  |  |
| No | 1 [Reference] |  |
| Yes | 0.72 (0.68–0.76) | <0.0001 |
| Gastrointestinal hemorrhage |  |  |
| No | 1 [Reference] |  |
| Yes | 0.82 (0.72–0.94) | 0.0037 |
| Covid19 pandemic |  |  |
| Before | 1 [Reference] |  |
| During | 0.94 (0.92-0.96) | <0.0001 |

Abbreviations: BT, bridging thrombolysis; DET, direct endovascular therapy; NIHSS, National Institutes of Health Stroke Scale; OR: Odds ratio.

FigureS1. Data source of CSPPC-R study


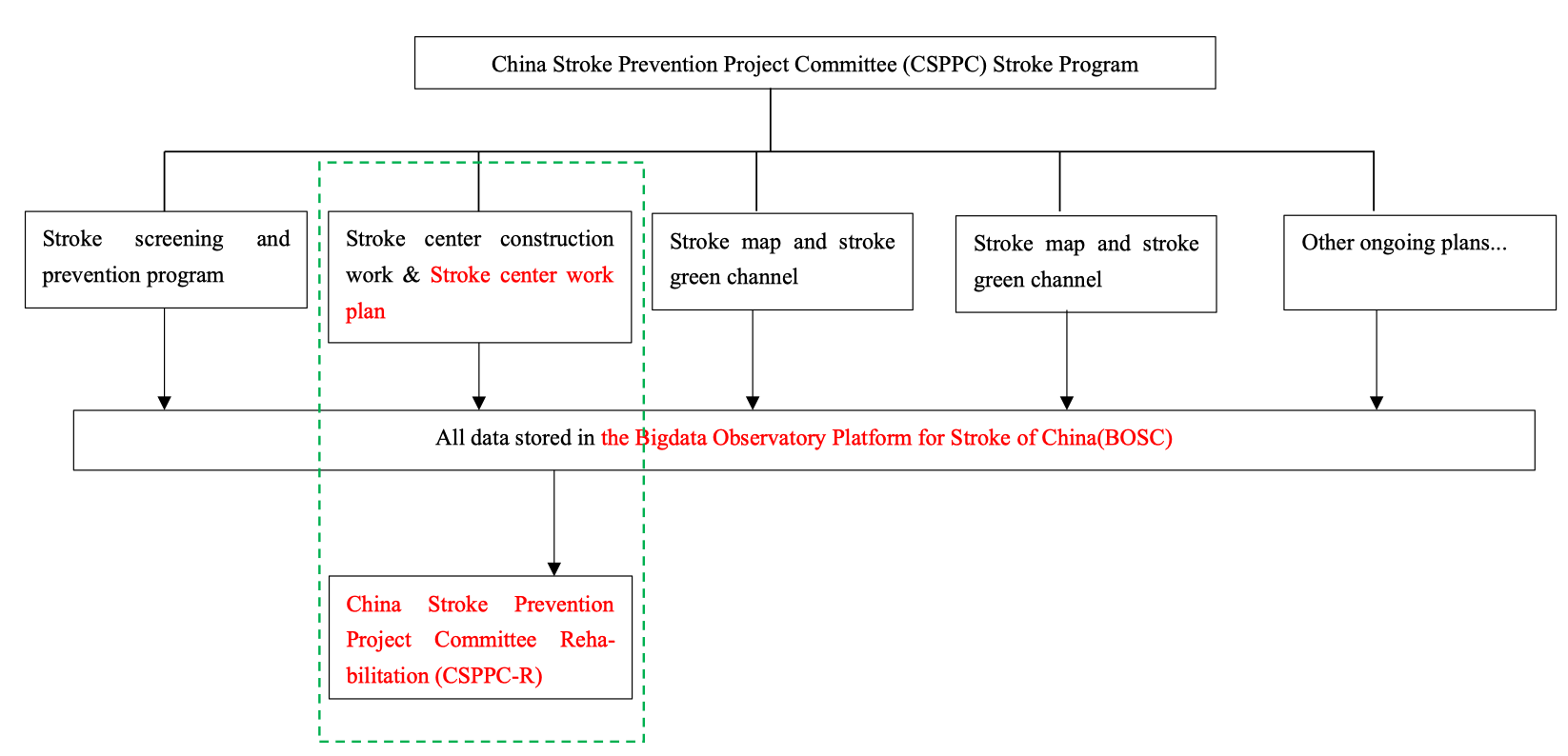


Note: All data of the CSPPC Stroke Program are collected and stored in the Bigdata Observatory Platform for Stroke of China(BOSC, https://www.chinasdc.cn)

References:

1. Chao BH, Yan F, Hua Y, et al. Stroke prevention and control system in China: CSPPC-Stroke Program. *Int J Stroke*. Apr 2021;16(3):265-72.

2. Shen Y, Chao BH, Cao L, et al. Stroke Center Care and Outcome: Results from the CSPPC Stroke Program. *Transl Stroke Res*. Jun 2020;11(3):377-86.

3. Chao BH, Tu WJ, Wang LD, Stroke Prevention Project Committee, and National Health Commission of the People’s Republic of China. Initial establishment of a stroke management model in China: 10 years (2011-2020) of Stroke Prevention Project Committee, National Health Commission. Chin Med J (Engl). 2021;134:2418-2420. doi: 10.1097/CM9.0000000000001856

FigureS2. Data of rehabilitation interventions and its numbers


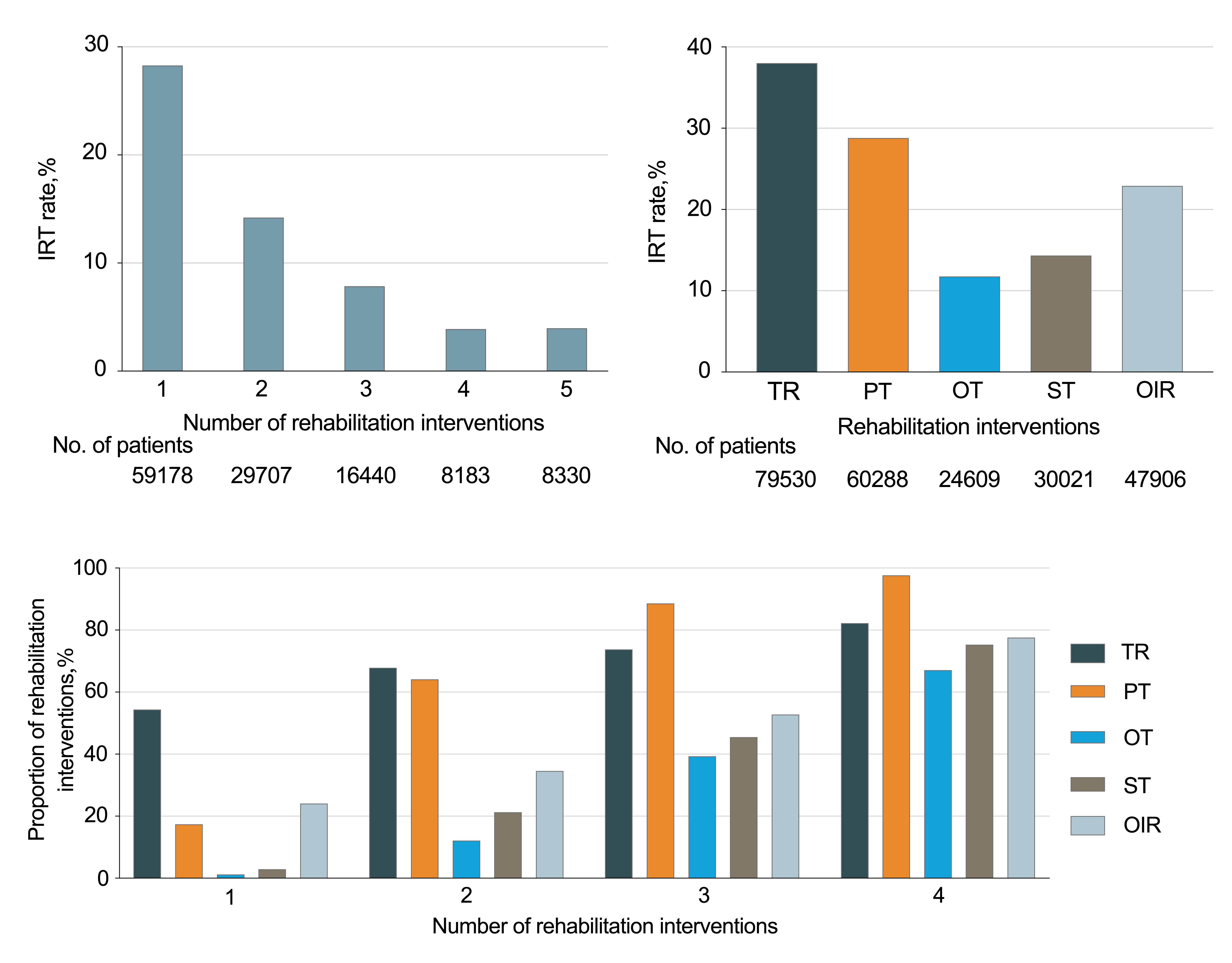


Abbreviations: IRT, inpatient rehabilitation therapy; OIR, other interventions of rehabilitation; OT, occupational therapy; PT, physical therapy; ST, speech therapy; TR, traditional rehabilitation.
